# Disentangling the root- and detritus-based food chain in the micro-food web of an arable soil by plant removal

**DOI:** 10.1371/journal.pone.0180264

**Published:** 2017-07-13

**Authors:** Olena Glavatska, Karolin Müller, Olaf Butenschoen, Andreas Schmalwasser, Ellen Kandeler, Stefan Scheu, Kai Uwe Totsche, Liliane Ruess

**Affiliations:** 1 Institute of Biology, Ecology Group, Humboldt-Universität zu Berlin, Berlin, Germany; 2 Institute of Soil Science and Land Evaluation, Soil Biology Department, University of Hohenheim, Stuttgart, Germany; 3 J.F. Blumenbach Institute of Zoology and Anthropology, Georg August University of Göttingen, Göttingen, Germany; 4 Institute of Geosciences, Hydrogeology Section, Friedrich Schiller University of JenaJena, Germany; 5 Centre of Biodiversity and Sustainable Land Use, University of Göttingen, Göttingen, Germany; University of Copenhagen, DENMARK

## Abstract

Soil food web structure and function is primarily determined by the major basal resources, which are living plant tissue, root exudates and dead organic matter. A field experiment was performed to disentangle the interlinkage of the root-and detritus-based soil food chains. An arable site was cropped either with maize, amended with maize shoot litter or remained bare soil, representing food webs depending on roots, aboveground litter and soil organic matter as predominant resource, respectively. The soil micro-food web, i.e. microorganisms and nematodes, was investigated in two successive years along a depth transect. The community composition of nematodes was used as model to determine the changes in the rhizosphere, detritusphere and bulk soil food web. In the first growing season the impact of treatments on the soil micro-food web was minor. In the second year plant-feeding nematodes increased under maize, whereas after harvest the Channel Index assigned promotion of the detritivore food chain, reflecting decomposition of root residues. The amendment with litter did not foster microorganisms, instead biomass of Gram-positive and Gram-negative bacteria as well as that of fungi declined in the rooted zone. Likely higher grazing pressure by nematodes reduced microbial standing crop as bacterial and fungal feeders increased. However, populations at higher trophic levels were not promoted, indicating limited flux of litter resources along the food chain. After two years of bare soil microbial biomass and nematode density remained stable, pointing to soil organic matter-based resources that allow bridging periods with deprivation. Nematode communities were dominated by opportunistic taxa that are competitive at moderate resource supply. In sum, removal of plants from the system had less severe effects than expected, suggesting considerable food web resilience to the disruption of both the root and detrital carbon channel, pointing to a legacy of organic matter resources in arable soils.

## Introduction

Soil organisms, their community structure and function in the food web, play a key role in soil carbon dynamics. However, while the turnover of total amounts and individual fractions of soil carbon are well investigated [1, 2, 3], less is known about the biotic diversity and interactions in soil food webs that determine major carbon and energy pathways [4, 5, 6]. Recent studies suggest that mineralisation and sequestration of carbon is shaped by the diversity within (horizontal diversity) and across (vertical diversity) trophic levels, and that the driving mechanisms are broadly the same across ecosystems [4]. This calls for experiments under field conditions to improve current food web models with empirical data and to disentangle the relationship between food web structure and ecosystem function [7].

Resource quality and availability, i.e. the horizontal diversity at the base of the food web, has an important impact on soil carbon flow [4, 6]. Soils comprise two major food chains, either root or detritus based [8]. In the root-based food chain the predominant carbon sources are living plant tissue and root exudates. In the detritus-based food chain dead organic matter fuels either the bacterial or fungal carbon channel, depending on labile or recalcitrant resources, respectively [9]. These differences in quality and accessibility of plant-derived substrates result in three major soil carbon and energy pathways based on roots, bacteria, and fungi [8, 10]. Particularly in arable systems the internal belowground carbon cycle is shaped by root-derived resources, due to the regular harvest of crop, and the variation in rhizodeposits depending on growing season [11].

Soil nematodes use an exceptionally wide range of resources and form functional groups at each trophic level of the food web [12], thereby holding a central position in both bottom-up and top-down controlled processes [13, 14]. Although some plant parasites cause disease, most taxa beneficially affect soil processes, such as nutrient mineralisation by bacteria [15, 16, 17]. In addition, nematode assemblages react quickly to shifts in availability and quality of exogenous resources [18, 19]. Due to these characteristics, nematodes are widely applied indicators for soil carbon pathways [9, 20]. Moreover, nematode life strategy and trophic composition reflect general food web conditions, expressed by the Enrichment and Structure Index calculated based on nematode family composition [21]. In arable land, the nematode faunal analysis concept was employed to indicate organic enrichment [22] as well as to assign changes in plant resources related to crop type, season or management practice [19, 23, 24]. Additionally, the effectiveness of fallow as method to control plant-parasitic nematodes and to restore soil properties has been investigated [25, 26, 27]. However, the impact of fallow or bare soil on soil food webs was rarely assessed [28].

To disentangle the root- and detritus-based food chain, a field experiment was performed on an arable land. By removal of plants the root channel was eliminated, and by organic amendment the detritus channel was fostered. Three treatments were established: plant (*Zea mays)* with removing stalks at harvest, application of maize shoot litter, and bare soil, providing roots, litter and autochthonous organic matter as predominant resources, respectively. These experimental manipulations separated the micro-food web into rhizosphere (maize plant), detritusphere (maize litter) and bulk soil (bare soil) habitats. While the rhizosphere food web comprises both the herbivore and the detritivore food chain, the detritusphere and the bulk soil food webs lack the herbivore chain, with bulk soil assemblages additionally facing deficiency of recent resource input.

Sampling at the field site was performed during two successive years, in July (high root exudation), September (plant residue input), and December (transport of organic matter), covering the major seasonal resource changes within a crop cycle. Microbial biomass and nematode fauna were investigated along a depth gradient from the topsoil (0–10 cm) to the rooted zone (40–50 cm) to root free soil (60–70 cm) (Kramer et al. [29]). We hypothesise that removing the plant from the system, i.e. the root based carbon and energy channel, will result in a loss in connectivity and complexity of soil food webs. Based on the predominant resources available the manipulations were expected to result in the following food web conditions: *i)* rhizosphere—highly structured with both herbivore and detrital food chain present, *ii)* detritusphere—moderately structured with detrital food chain solely, and *iii)* bulk soil—low structure of assemblages inhabiting bare soil.

## Materials and methods

### Field site and agriculture management

The experiment was conducted on an arable field near Göttingen (Germany), a region with a mean annual precipitation of 720 mm and mean air temperature of 7.9°C. The dominant soil types at the site are Cambisols and Luvisols, with a pH (CaCl_2_) of 6.0, a bulk density of 1.4 g cm^-3^, an organic C and a total N content of 12.4 and 1.3 g kg^-1^, respectively. More details on physical and chemical soil properties are given in Kramer et al. [29]. The field work at Reinshof Experimental Farm has been permitted by the University of Göttingen.

During the spring (April 2012), the non-selective herbicide Round-up (Glyphospate: 4 l ha^-1^) was applied to the field followed by tillage of the land using a chisel plough to a depth of 12 cm. The maize cultivar Codosco (I.G. Pflanzenzucht GmbH, München, Germany) was then sown at a density of 11.5 grains m^-2^. Inorganic N fertilizer (ammonium nitrate urea solution: 76 kg N ha^-1^, ammonium sulphate: 20 kg N ha^-1^) and NP fertilizer (diammonium phosphate: 19 kg N ha^-1^, 111 kg P ha^-1^) were applied shortly prior to and after sowing of the maize seeds to improve plant growth. During the growing season the plots received a combination of herbicides: Peak: 20 g ha^-1^ (750 g kg^-1^ prosulforon, for control of broad leaved weeds in forage and grain maize), EFFIGO: 0.35 l ha^-1^ (267 g l^-1^ clopyralid + 67 g l^-1^ picloram, a post-emergence herbicide to control dicotyledonous weeds), MILAGRO: 0.50 l ha^-1^ (240 g l^-1^ nicosulfuron, for the control of grass in forage and grain maize), Terbuthylazin 500: 0.85 l ha^-1^ (a selective residual triazine herbicide for pre and post emergent weed control in maize). At harvest in September 2012, the corncobs were removed by hand and maize plants were cut to a height of 10 cm above soil surface. Maize stalks were then shredded to particle sizes of < 1 cm^2^ and air-dried to obtain maize litter.

Again, in the following spring (April 2013) Glyphosate (4 l ha^-1^; N-phosphonomethyl-glycine, a post-emergence, non-selective, foliar herbicide) was used to kill the layer of weeds that had developed. Three weeks later, the soil was tilled to a depth of 12 cm and the maize cultivar Codosco planted at a density of 8.5 grains m^-2^ and fertilized according to the practice in 2012. The same herbicides were applied, with slight variations accounting for the emergence of weeds, in the following combinations: Terbuthylazin 500: 0.85 l ha^-1^, MILAGRO: 0.50 l ha^-1^, Peak: 20 g ha^-1^, EFFIGO: 0.35 l ha^-1^. The crop was harvested in September (2013) and all crop residues removed from the experimental field site.

### Treatments

In May 2012, a total of 12 experimental plots (size 5 x 5 m) were established on the field in two adjacent rows separated by a 5 m buffer stripe within and 2 m buffer strips between rows. Three treatments were assigned to the plots on the basis of the different resource qualities for the soil food web: plant (maize as crop), litter (application of maize shoot litter) and bare soil, each with four replicates. In the plant treatment, which constituted all the major food web resources, there is a supply of carbon via living root tissues and rhizodeposition carbon during the growing period and after the harvest decaying roots continue to provide belowground carbon. Litter and bare soil treatments were established by allowing maize plants to grow for three weeks after sowing and then removing the plants by hand from the plots. Therafter, for the litter treatment the resource for the detritus-based food web was enhanced by amendment with shredded maize shoot litter applied to the soil surface at an amount of 0.8 kg dry weight m^-2^ (equivalent to 0.35 kg C m^-2^), resembling the shoot biomass of maize crop. In the bare soil treatment the food web did not receive any recent plant resources and was therefore dependent on autochthonous soil organic matter.

Litter and bare soil treatment plots were covered by nets (AGROFLOR Kunststoff GmbH, Wolfurt, Austria) to adjust for differences in light intensity and temperature between planted and unplanted plots, during maize growing period. Shading was set up at a level representing mean leaf area index (LAI) of plants during the vegetation period, which was compared randomly by a lux meter across plots. In addition, weeding of all plots was performed at regular intervals across seasons to maintain experimental treatments and to prevent plant carbon input by weeds.

### Soil water regime

In August 2012, six EnviroSCAN water content profile probes (Sentek Inc., Sidney, Australia) were installed in three plots that cover each treatment with two replicates to determine soil moisture conditions at the field site. In the plant plots, the first sensor was installed directly in a crop row and the second between the rows. The volumetric water content of the soil was determined at four depths (0–10, 10–20, 20–30 and 40–50 cm) from August 2012 until June 2013, thereafter measurements were aborted due to the theft of data loggers. Twelve tension-controlled lysimeters (UMS GmbH, München, Germany) were installed in six plots at two depths to monitor water and organic matter fluxes below the plough layer (30 cm) and below the main rooting zone (60 cm). Seepage water samples were taken every fortnight from August 2012 until April 2014 [30].

A compensation of water uptake by the plants via conventional irrigation was initially intended but turned out not to be feasible due to the very variable temporal and spatial soil moisture distribution at planted plots. Relevant differences in soil water content with maize presence occurred only in autumn, where predominantly the subsoil below the plough layer was dryer, whereas in summer the planted plots were wetter in topsoil and rooted zone (S1 Table). Soil water contents of the different treatments converged in winter with those under bare soil at all plots.

### Sampling

Soil samples were collected during two successive vegetation periods of 2012 and 2013, in July, September, and December. Samples were taken at three different depths: plough layer (0–10 cm), rooted zone (40–50 cm) and root free zone (60–70 cm). From each plot eight samples were taken with a soil corer (diam. 2.5 cm), bulked and gently mixed by hand. Subsamples of about 50 g fresh weights each were taken for analysis of microorganisms and nematodes. An additional 30 g fresh weight was used for determination of the actual soil water content at sampling date. For each treatment and soil depth four replicates were collected resulting in a total of 216 samples during the two seasons.

### Microorganisms

Phospholipid fatty acids (PLFAs) were extracted from 2 x 4 g and 2 x 10 g of each topsoil and subsoil samples, respectively, using a Bligh and Dyer solution (chloroform, methanol, citrate buffer (pH 4), 1:2:0.8, v/v/v) as described by Frostegård et al. [31]. Fractionation into glycolipid, neutral lipid and phospholipid fatty acids was performed using silica acid columns (Bond Elut SI, 500 mg, 3 ml, Agilent Technologies Inc, Santa Clara, USA). The two replicates of each sample were combined into one column before fractionation. To transform PLFAs into fatty acid methyl esters (FAMEs), an alkaline methanolysis was done as described by Ruess et al. [32].

The FAMEs were measured with an AutoSystem XL gas chromatograph (Perkin-Elmer Corporation, Norwalk, CT, USA) equipped with a capillary column HP-5 (50 m × 0.2 mm, film thickness of 0.33 μm) and a flame ionization detector. Helium was used as carrier gas. The temperature program started with 70°C for 2 min, increased with 30°C min^-1^ to 160°C, further 3°C min^-1^ to 280°C and held for 15 min. The injection temperature was 260°C.

The total amount of soil PLFAs was used as proxy for the total microbial biomass. The share of Gram-positive bacteria was assigned by their specific marker PLFAs i15:0, a15:0, i16:0 and i17:0, and that of Gram-negative bacteria by the markers cy17:0 and cy19:0, whereas 18:2ω6,9 was used for saprotrophic fungi [31, 33]. Please note that total soil PLFAs comprise, besides microorganisms, also fatty acids derived from other organisms and tissue. However, the pool size of bacteria and fungi in soil PLFAs is much higher compared e.g. plants and animals, as the latter predominantly contribute fatty acids to the soil neutral lipid fraction [33].

### Nematode fauna

Nematodes were extracted from 50 g of soil per sample using a modified Baermann method according to [34]. The extraction was at room temperature for 24 h (approx. 18°C), followed by a heating regime with successive 5°C steps for 6 h, from 20°C to 45°C. Nematodes were fixed in 4% cold formaldehyde solution. For each sample the total number of nematodes was counted under a light microscope at 63×magnification, and then 10% of the total individuals per sample were determined to genus level at 630×magnification.

As a scale of soil ecosystem conditions nematode faunal analysis was applied without considering the dauerlarvae (non-active stage) of Rhabditidae. This concept is based on nematode life history strategy and distinguishes families into colonizers (*c*) and persisters (*p*) as extremes on a scale from 1 to 5, respectively [35]. To determine the major carbon channels, food web enrichment and structure the following indices were assigned: the ratio of fungal to bacterial feeder (*F/B)* [36] and the Enrichment (*EI*), Structure (*SI*), Basal (*BI*) and Channel Index (*CI)* according to Ferris at al. [21]. The *F/B* ratio mirrors changes in nematode microbial resources and the *CI* determines the dominance of the fungal or bacterial energy channel in the soil food web. The *EI* provides information on nutrient availability and soil fertility, while the *SI* informs about the stability and structure of the soil food web and the regeneration stage after a disturbance. The following equations were used: EI = 100 * e / (e + b); SI = 100 * s / (s + b), BI = 100*b/ (e + s + b) and CI = 100 * (0.*8 Fu*_*2*_ */ (3*.*2Ba*_*1*_ *+ 0*.*8Fu*_*2*_*)*, where *e* is the enrichment, *b* the basal, and *s* the structure component. *Fu*_*2*_ represents fungal feeders with *c-p*-classification 2, *Ba*_*1*_ bacterial feeders with *c-p*-classification 1.

### Statistical analysis

The effects of major resource, time and soil depth on nematode communities (i.e. density, taxa, indices) and on PLFA amounts of total microorganisms, bacteria, and fungi, were analysed by analysis of variance (ANOVA). Microbial data were log transformed, and data sets that achieved normal distribution were subjected to further analyses. As both microbial biomass and nematode density decreased significantly with depth, and moreover soil horizons along the profile are dependent, each soil layer was investigated separately. A repeated-measures ANOVA for each vegetation period with the factors season (S) and treatment (T) was performed. Differences between means with one sampling date were inspected using Tukey's Honestly Significant Difference (HSD) test. Analysis was performed using STATISTICA 9.1 for Windows (StatSoft, Hamburg).

The Spearman's rank-order correlation was employed to assign the association between nematodes and biotic (microorganisms) and abiotic variables (soil moisture). This nonparametric correlation was employed, as data for soil water content did not achieve normal distribution even after transformation. The Spearman's correlation determines the strength and direction of the relationship between two variables, here nematode trophic groups and microbial diet, and nematode density and actual soil water content at sampling.

## Results

### Biomass of microorganisms

**Fig 1:**
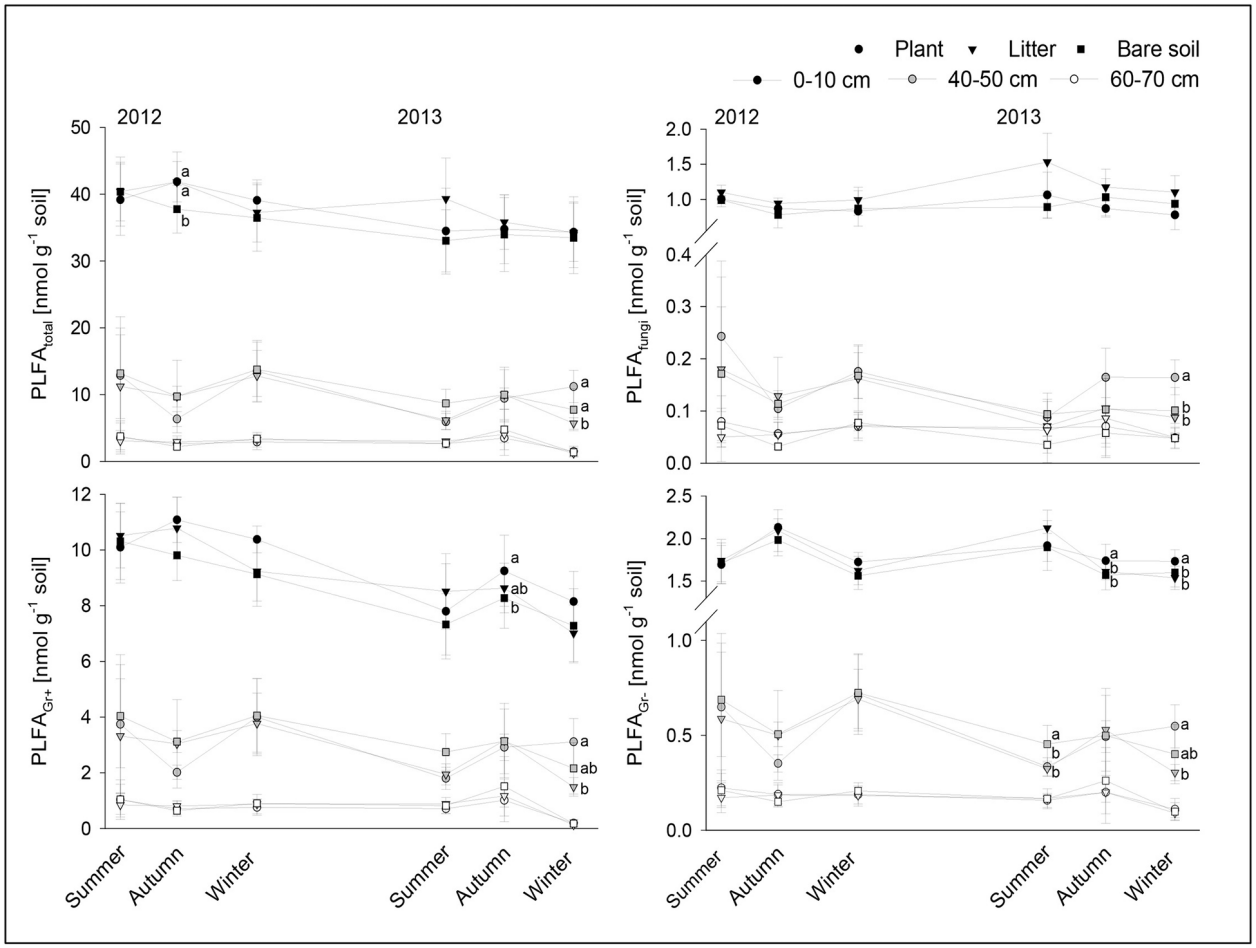
Biomass of microbial groups determined as soil phospholipid fatty acids (PLFAs in nmol g^-1^ dry weight soil ± SD) in soil cropped with maize (plant), amended with maize shoot litter (litter) or bare soil, in topsoil (0–10 cm), rooted zone (30–40 cm), and root free zone (60–70 cm) in the years 2012 and 2013. Given is the biomass for total microbial assemblages (PLFA_total_), Gram-positive bacteria (PLFA_GR+_), Gram-negative bacteria (PLFA_GR-_) and fungi (PLFA_fungi_). Values within a sampling date with the same or no letters are not significantly different according to Tukey´s HSD test at *P* < 0.05.

Based on the marker PLFAs detected, Gram-positive bacteria were the dominant microbial group across treatments and depth (Fig 1). Treatment effects on the different microbial groups occurred predominantly in the second vegetation period and were restricted to the topsoil and the rooted zone. In the topsoil the total microbial biomass increased in plant and litter plots compared to bare soil plots in autumn 2012 (*F*_*2*,*9*_ = 4.797, *P* = 0.043). Plant presence further enhanced the biomass of Gram-positive bacteria in autumn (*F*_*2*,*9*_ = 4.919, *P* = 0.040), and of Gram-negative bacteria in autumn (*F*_*2*,*9*_ = 6.080, *P* = 0.025) and winter (*F*_*2*,*9*_ = 12.028, *P* = 0.004), in comparison to litter and bare soil plots in 2013. In contrast, in litter amended plots there was a tendency for fungal biomass to increase during summer (*F*_*2*,*9*_ = 3.907, *P* = 0.066) and winter (*F*_*2*,*9*_ = 4.438, *P* = 0.051) as compared to the bare soil and plant plots.

During the first vegetation period no changes were detected in the rooted zone, whereas during the second year, the biomass of total microorganisms, fungi, Gram-positive and Gram-negative bacterial were affected by treatments in winter (PLFA_total_: *F*_*2*,*9*_ = 8.120, *P* = 0.012; PLFA_fungi_: *F*_*2*,*9*_ = 12.982, *P* = 0.003; PLFA_Gr+_: *F*_*2*,*9*_ = 6.870, *P* = 0.018; PLFA_Gr-_: *F*_*2*,*9*_ = 7.234, *P* = 0.016), with plant plots displaying highest, while litter amended and bare soil plots lowest and intermediate values, respectively. The Gram-negative bacteria also responded earlier in the season (summer: *F*_*2*,*9*_ = 4.559, *P* = 0.048) with highest biomass at bare soil plots.

### Nematode density and community structure

**Fig 2:**
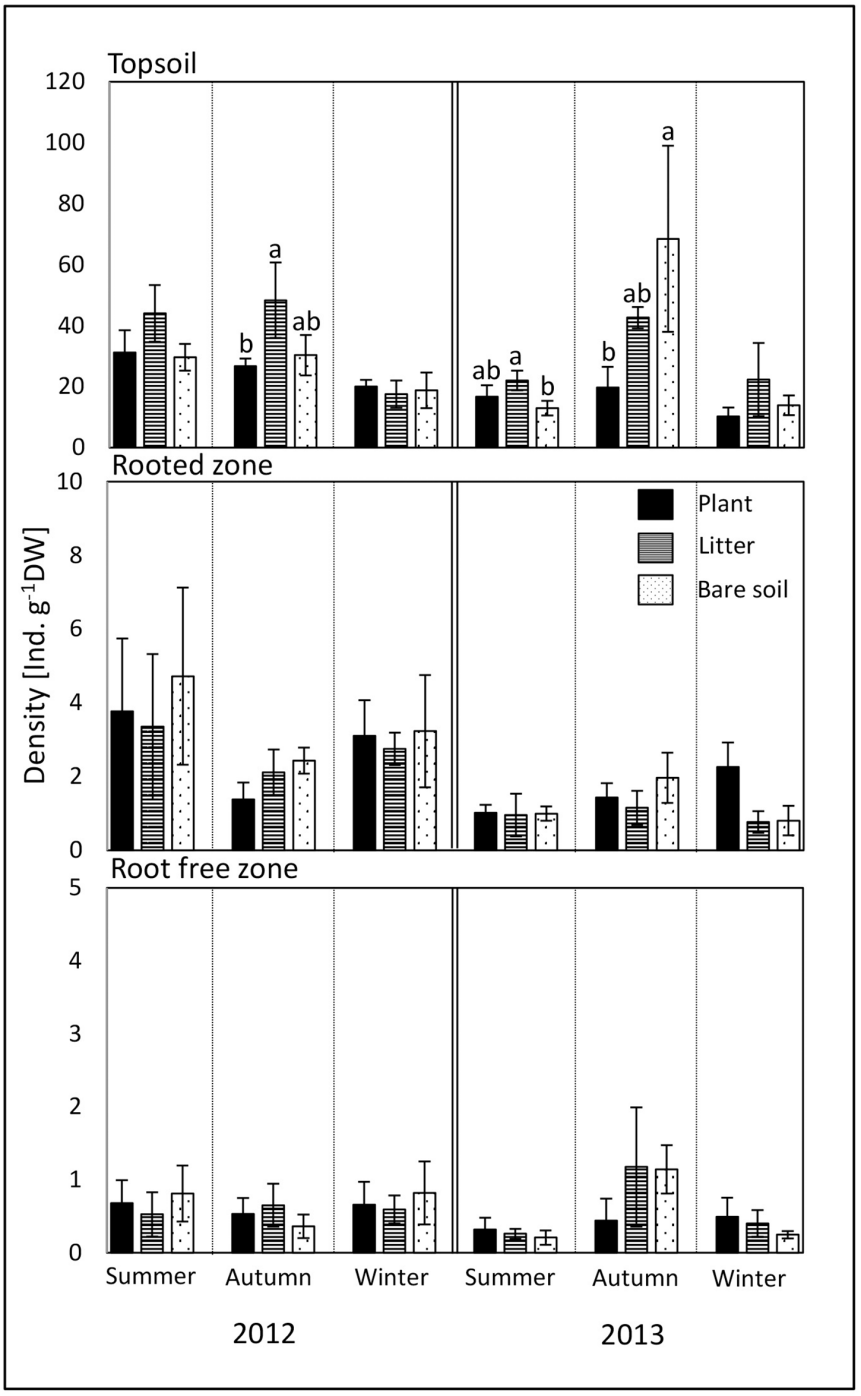
Density (individuals g^-1^ dry weight soil ± SD) of nematodes in soil cropped with maize (plant), amended with maize shoot litter (litter) or bare soil, in topsoil (0–10 cm), rooted zone (40–50 cm), and root free zone (60–70 cm) in the years 2012 and 2013. Values within a sampling date with the same or no letters are not significantly different according to Tukey´s HSD test at *P* < 0.05.

Significant effects of the different treatments on nematode population density were restricted to the topsoil (Fig 2). The amendment with litter fostered the density in summer and autumn across both years (ANOVA, *P* < 0.05). The absence of plants had no negative impact on nematode density and more nematodes were detected under bare soil compared to plant plots in autumn 2013 (Tukey´s HSD test, *P* < 0.05).

**Fig 3:**
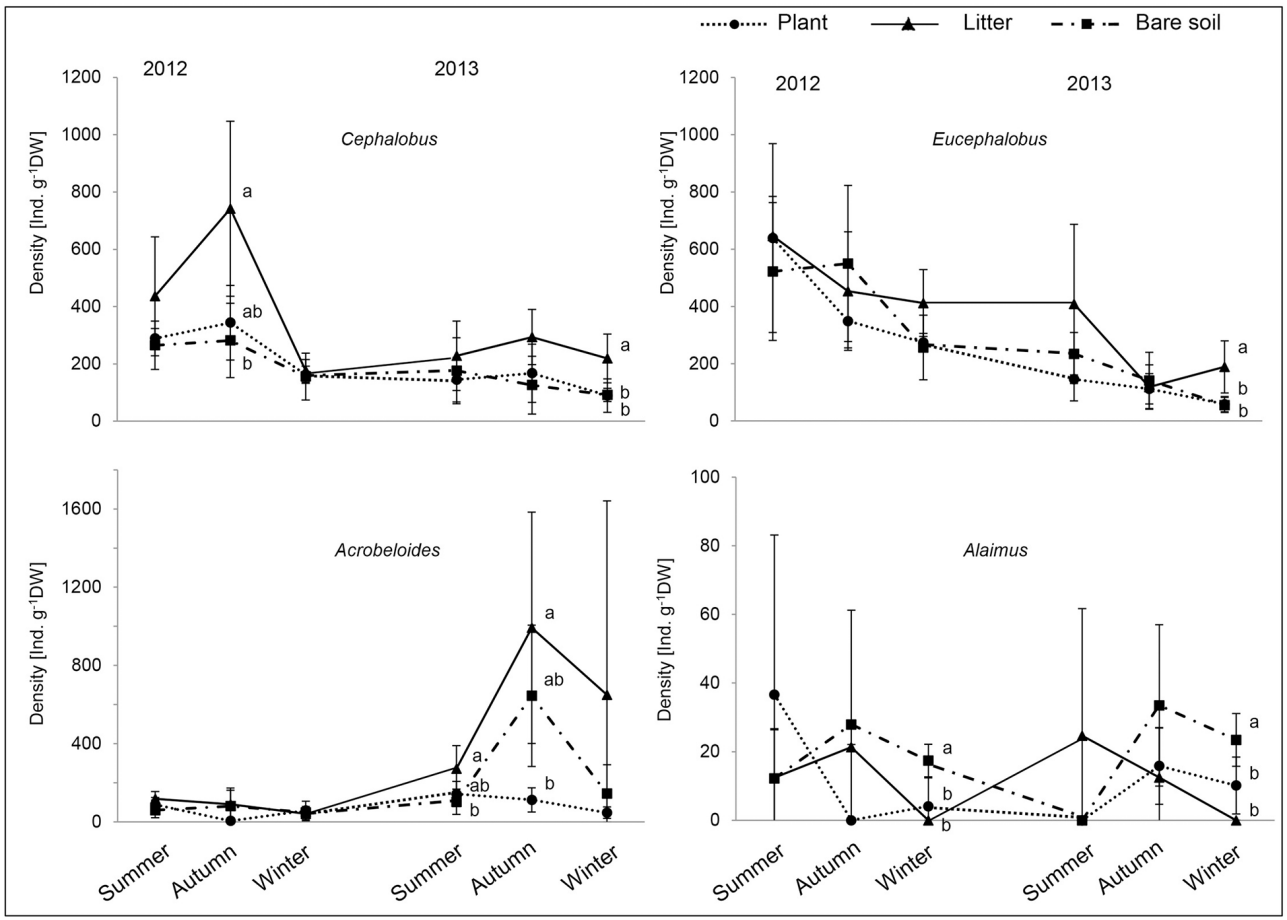
Occurrence of the bacterial-feeding taxa *Cephalobus*, *Eucephalobus*, *Acrobeloides* and *Alaimus* in soil cropped with maize (plant), amended with maize shoot litter (litter) or bare soil. Abundance (individuals 100 g^-1^ dry weight soil ± SD) is given in topsoil (0–10 cm), rooted zone (40–40 cm), and root free zone (60–70 cm) in the years 2012 and 2013. Values within a sampling date with the same or no letters are not significantly different according to Tukey´s HSD test at *P* < 0.05.

**Fig 4:**
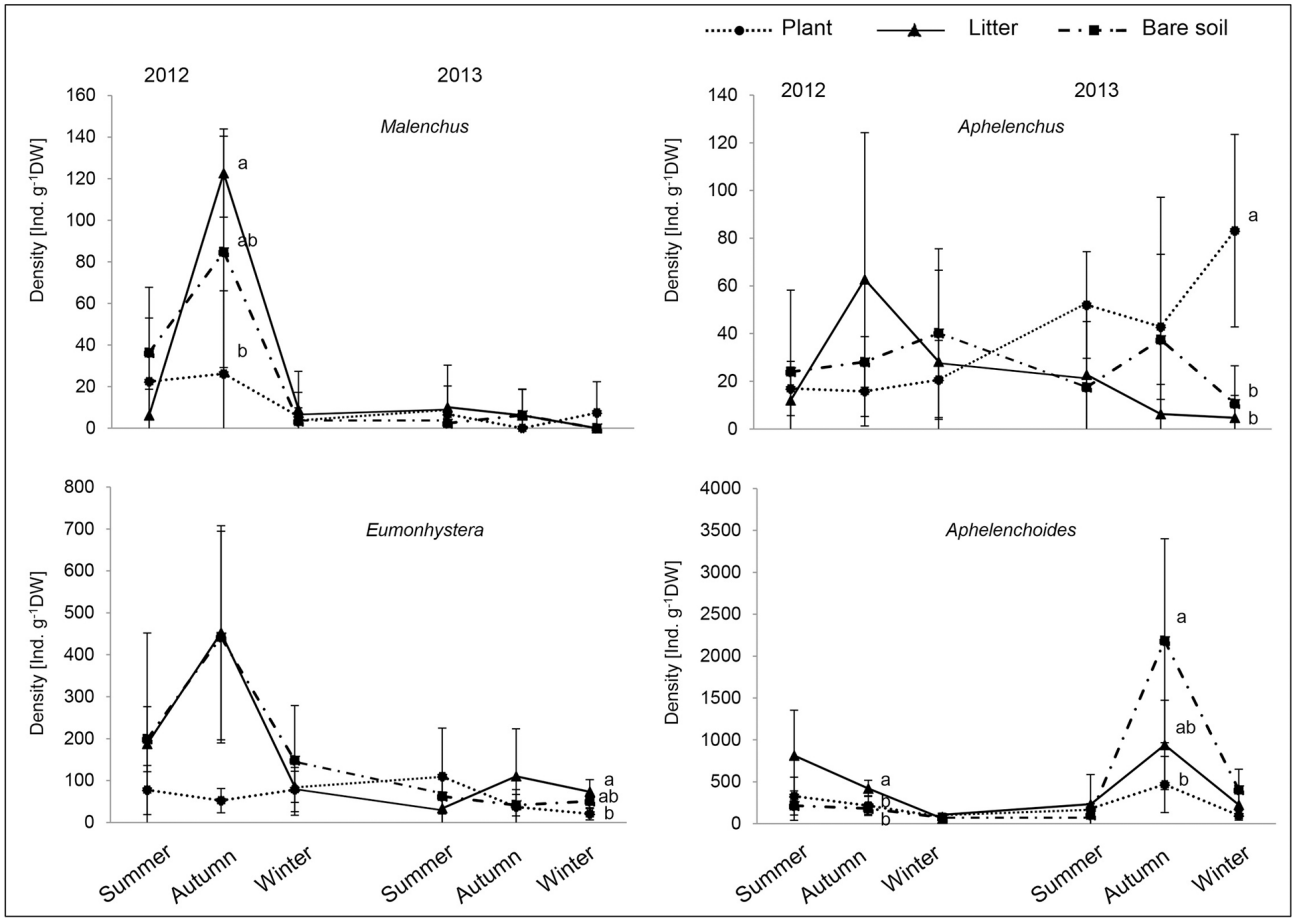
Occurrence of the root-feeding *Malenchus*, the bacteria and unicellular eukaryote feeding *Eumonhystera*, and the fungal-feeding *Aphelenchus* and *Aphelenchoides* in soil cropped with maize (plant), amended with maize shoot litter (litter) or bare soil. Abundance (individuals 100 g^-1^ dry weight soil ± SD) is given in topsoil (0–10 cm), rooted zone (40–50 cm), and root free zone (60–70 cm) in the years 2012 and 2013. Values within a sampling date with the same or no letters are not significantly different according to Tukey’s HSD test at *P* < 0.05.

Comparing the abundance of frequent bacterial feeders with regular occurrence across seasons in the topsoil revealed quite different patterns (Fig 3). *Cephalobus* was facilitated by litter predominantly during the first year (autumn 2012: *F*_2,9_ = 5.88, *P* = 0.023, winter 2013: *F*_2,9_ = 5.91, *P* = 0.023), whereas the related genus *Acrobeloides* showed an opposite density distribution (summer 2013: *F*_2,9_ = 4.27, *P* = 0.049, autumn 2013: *F*_2,9_ = 4,87, *P* = 0.037). The abundance of *Eucephalobus* decreased over the experimental period with a positive impact of litter amendment in winter 2013 (*F*_2,9_ = 7.18, *P* = 0.01), whereas *Alaimus* proliferated under bare soil at both winter samplings (winter 2012: *F*_2,9_ = 11, *P* = 0.004, winter 2013: *F*_2,9_ = 12.95, *P* = 0.002; Fig 4). *Eumonhystera*, a genus feeding mainly on bacteria but also ingesting unicellular eukaryotes, had its highest density in litter and bare soil plots in autumn of the first year, however, a significant treatment effect was only visible during winter 2013 under litter compared to plant treatment plots (*F*_2,9_ = 6.13, *P* = 0.021, Fig 4). A similar pattern occurred for the root-feeding *Malenchus* (autumn 2012: *F*_2,9_ = 5.53, *P* = 0.027). Among fungal feeders *Aphelenchoides* showed a variable distribution pattern with positive response to litter in the first (autumn 2012: *F*_2,9_ = 6.21, *P* = 0.025) as well as to the bare soil treatment in the second season (autumn 2013: *F*_2,9_ = 5.02, *P* = 0.03), whereas plant presence promoted the occurrence of *Aphelenchus* with time, which was significant in winter 2013 (*F*_2,9_ = 11.64, *P* = 0.003).

### Nematode trophic structure and faunal indices

**Fig 5:**
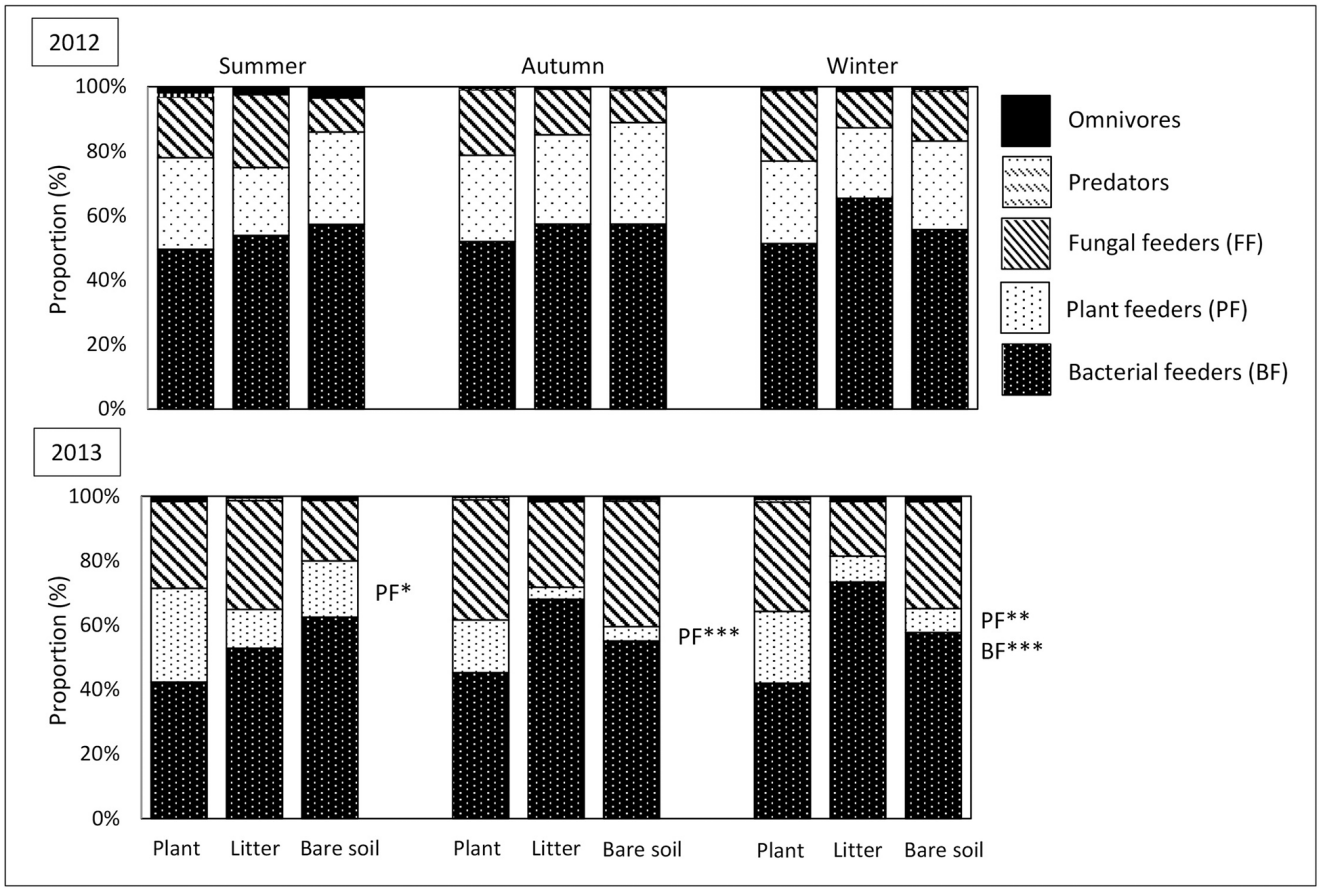
Proportion of nematode trophic groups (% ± SD) in soil cropped with maize (plant), amended with maize shoot litter (litter) or bare soil in topsoil (0–10 cm) in the years 2012 and 2013. ANOVA with the factors season (S) and treatment (T), significant effects are indicated by *, **, *** at *P* < 0.05, 0.01, 0.001.

The major trophic groups of nematodes across treatments were bacterial feeders with an average relative abundance of 56%, followed by fungal (23%) and plant (20%) feeders in the upper soil layer (Fig 5). Omnivores and predators were scarce with proportions below 1.1%. While no significant differences were detected during the first plant growth period, in the second season the proportion of plant feeders strongly declined in the absence of a plant (summer: *F*_2,9_ = 4.19, *P* = 0.05, autumn: *F*_2,9_ = 8.29, *P* = 0.009 and winter: *F*_2,9_ = 5.03, *P* = 0.034), whereas the proportion of bacterial feeders was distinctly higher in litter and bare soil plots in the topsoil (winter: *F*_2,9_ = 14,29, *P* = 0.0016).

The occurrence of nematode trophic groups was related to the biomass of microorganisms at the base of the food web (S2 Table). In particular, bacterial feeders showed positive correlations (*P* < 0.04) to Gram-positive and Gram-negative bacteria across seasons (except winter 2012 and summer 2013), however this was restricted to the rooted zone in 40–50 cm depth (S2A and S2B Table). A similar pattern occured for plant feeders and bacteria (*P* < 0.04, not summer and autumn 2013), with additional positive relations between these groups at 60–70 cm depth during summer 2012 and autumn 2013. In contrast, the frequency of fungal feeders was not linked to fungi (S2C Table). For higher trophic level nematodes, omnivores showed positive correlations in the topsoil (winter 2013, *P* < 0.04) and rooted zone (summer and autumn 2012, *P* < 0.01) with all microbial groups, whereas for predators this relationship was weak with only once showing a negative correlation to Gram-negative bacteria (autumn 2012, *R* = -0.72, *P* = 0.01; S2A–S2C Table).

In the first plant growing season no distinct changes in micro-food web conditions were assigned by nematode faunal analyses. In the second vegetation period the average Enrichment Index (*EI*) in the upper soil layer was 58, indicating a moderate to good nutrient availability (Table 1). The *EI* was higher at the plant compared to litter plots, pointing to nitrogen enrichment during the growing season (summer 2013, Tukey’s HSD test, *P* < 0.05). The Structure Index (*SI*), ranging from zero to 30, indicated disturbance and very low food web complexity, but no treatment effects. In contrast, the Basal Index (*BI*) was affected by plant presence and increased in the rooted zone in summer and winter, whereas it decreased in the topsoil during the summer (Tukey’s HSD test, *P* < 0.05). The Channel Index (*CI*) with an average below 50 indicated a carbon flow mainly through the bacterial channel, with the highest values at the plant plots in summer and winter (Tukey’s HSD test, *P* < 0.05).

**Table 1:** Nematode food web conditions (Community Indices ± SD) at plots cropped with maize (plant), amended with maize shoot litter (litter) or bare soil, in topsoil (0–10 cm), rooted zone (40–50 cm) and root free zone (60–70 cm) after two vegetation periods.

Depth	Indices	Summer			Autumn			Winter			ANOVA
		Plant	Litter	Bare soil	Plant	Litter	Bare soil	Plant	Litter	Bare soil	
0–10 cm	*EI*	53 ± 6**a**	41 ± 6**b**	45 ± 1**ab**	59 ± 9	69 ± 8	77 ± 11	58 ± 5	59 ± 15	68 ± 10	S***, SxT*
	*SI*	11 ± 4	11 ± 9	6 ± 5	7 ± 6	11 ± 2	13 ± 12	17 ± 9	7 ± 12	22 ± 6	
	*BI*	45 ± 5**b**	55 ± 5**a**	53 ± 1**ab**	40 ± 9	30 ± 8	22 ± 11	39 ± 4	40 ± 16	29 ± 9	S***, SxT*
	*CI*	43 ± 15	56 ± 25	30 ± 19	43 ± 25	18 ± 6	24 ± 15	40 ± 9**a**	18 ± 9**b**	26 ± 12**ab**	S*
40–50 cm	*EI*	40 ± 10	51 ± 11	58 ± 7	57 ± 6	67 ± 10	63 ± 5	55 ± 9**b**	77 ± 11**a**	61 ± 10**ab**	S***, T*
	*SI*	7 ± 13	13 ± 15	0	0	10 ± 9	7 ± 8	6 ± 8	30 ± 36	11 ± 13	T*
	*BI*	57 ± 10**a**	44 ± 5**ab**	42 ± 7**b**	43 ± 6	31 ± 9	36 ± 6	44 ± 10**a**	22 ± 12**b**	37 ± 11**ab**	S**, T*
	*CI*	88 ± 15**a**	63 ± 27**ab**	45 ± 13**b**	47 ± 24	34 ± 13	43 ± 13	57 ± 18**a**	17 ± 8**b**	33 ± 17**ab**	S***, T*
60–70 cm	*EI*	61 ± 12	63 ± 32	39 ± 20	75 ± 12	76 ± 10	76 ± 11	52 ± 22	57 ± 9	55 ± 8	S*
	*SI*	0	0	0	0	28 ± 29	10 ± 12	10 ± 20	10 ± 20	0	
	*BI*	39 ± 12	37 ± 32	61 ± 20	25 ± 12	22 ± 11	23 ± 11	42 ± 13	39 ± 2	45 ± 8	S**
	*CI*	60 ± 35	52 ± 55	71 ± 34	24 ± 18	21 ± 12	18 ± 8	69 ± 38	22 ± 16	56 ± 31	S*

Presented are the *EI*–Enrichment Index, *SI*–Structure Index, *BI*–Basal Index, and *CI*–Channel Index. ANOVA with the factors season (S) and treatment (T) with *, **, *** as *P* < 0.05, 0.01, 0.001. Values within a row and sampling date with the same or no letters are not significantly different according to Tukey’s HSD test at *P* < 0.05.

## Discussion

### Rhizosphere food web

The rhizosphere associated food web comprises both, the herbivore and detritivore food chain, and therefore represent a hotspot of species interactions [37, 38, 39]. The presence of maize plants had a positive impact on microorganisms, and total biomass increased in the first, and that of all groups (total, Gram-positive and Gram-negative bacteria, fungi), in the second season. These effects occurred in autumn and winter, yet were not apparent in the root free zone. Previous studies at the experimental field site estimated that maize allocated 0.30 kg C m^-2^ as root-C belowground [40]. During fallow periods primary decomposers obviously rely on such root carbon of the harvested crop. This is supported by the increase in the fungal decomposition channel assigned by the higher *CI* in nematode communities, indicating usage of recalcitrant (e.g. dead roots) resources.

A growing plant sustains energy flux belowground via both, the primary production-based herbivore and the decomposition-based detrital food chain. As in microorganisms (see above), this dual resource supply likely fosters nematode population development in comparison to the litter and bare soil treatments. However, nematode numbers at plant plots were generally similar to those under bare soil in the topsoil, and no positive plant impact in the rooted and root-free zone was observed. This could be attributed to the reduced soil moisture due to water uptake by plants. Both Görres et al. [41] and Alphei et al. [42] observed a distinct effect of soil water content on nematodes density. During the experimental period the seasonal average of the soil moisture showed only small differences, ranging between 24 to 32% (volumetric water content) across all treatments and seasons (S1 Table). In summer, the planted soil was slightly wetter in the topsoil and rooted zone, whereas in autumn it was dryer. However, this had not negative impact on nematode density, which corresponds to Griffiths et al. [43] showing that variations sufficient to affect plant growth had only minor impact on the nematode community.

Another reason for the lack in a general promotion of the micro-food web by maize presence could be its low root biomass with 0.08 to 0.2 mg C g^-1^ soil only [40]. From 2009 to 2012 the arable field site was cultivated by wheat, with twice as much root biomass in the topsoil [29], beneficially affecting microbial as well as meso- and macrofauna communities as compared to maize [44, 45, 46]. Among nematodes the decline of the plant-feeding *Malenchus* and the rhizosphere associated bacterial-feeding *Eucephalobus* [47] with time also points to maize as a poor host. However, in the second season the density of plant feeders was highest at plants plots, predominantly in summer, which coincides with the maximum development stage of maize roots [40]. Overall, the herbivore food chain was clearly promoted under maize crop compared to litter and bare soil plots.

As herbivore and detritivore food chains merge at higher trophic levels, plant presence can support a more diverse food web with a considerable build-up of higher trophic levels [48]. However, in the investigated arable soil, the density of predators and omnivores was low, also reflected by the low *SI*, pointing to a disturbed and basal food web across all treatments [49]. Thus, the enhanced resource entry into the micro-food web at plant plots was not mirrored by higher trophic levels, suggesting that the energy flux along the food chain was generally low. Correspondingly, Zhang et al. [50] reported that bottom-up effects of vegetation on plant-feeding nematodes were weakly correlated with predator occurrence. In sum, the structure of the rhizosphere micro-food web at the plant plots, although characterized by high resource availability, was set by the predominance of bottom-up effects to lower trophic levels but this did not result in more connectivity, i.e. energy flux and biomass build up, to higher trophic levels.

### Detritusphere food web

Compared to rhizosphere communities, food webs in bulk soil, based on detrital resources only, were assumed to be less complex. By adding maize litter the resource supply to the detritus based food chain was enhanced by a rate of 0.35 kg C m^-2^. In previous studies at the experimental field similar amendment fostered microorganisms, in particular fungi and the fungal decomposition channel, yet these plots were cultivated with maize or wheat during the vegetation period [44, 45, 51]. In the present experiment with the root channel excluded, application of maize litter did not alter total microbial biomass significantly as compared to the bare soil treatment, except once in autumn of the first season in topsoil. These findings highlight the importance of the linkage between the herbivore and detritivore food chain, i.e. food web connectivity and structure, for the energy transfer within the entire food web.

During the second year the biomass of all microbial groups was generally lowest in the rooted zone of the litter plots. In contrast, the nematode density was high, reflecting the input of organic matter, which is reported to increases total numbers of nematodes [22, 52, 53]. This points to enhanced nematode grazing as regulating agent for microbial biomass, and is supported by the positive correlation of bacterial biomass and bacterial feeders. The amendment of soil with recalcitrant plant residues generally results in enhanced carbon incorporation into the fungal food chain [45, 54]. However, enhanced carbon flux in the fungal decomposition channel was not assigned by the *CI* at litter plots. The maize shoot litter applied was rich in nitrogen (C to N ratio 18.3); furthermore maize litter contains large amounts of easily decomposable carbohydrates (O-alkyl-C as 79% of shoot biomass) [55]. This may explain the observed high activity of the bacterial channel in the detritusphere food web, which was most apparent during winter across soil depths. At that season bacterial populations were mobilized and transported along the soil depth profile at the experimental arable field [30].

Within nematode communities, plant-feeders showed considerable resilience to the absence of plants, with negative effects only in the second season. They were mainly represented by the family Tylenchidae, taxa generally assigned as plant parasites [12] or “plant associated” [56, 57], but fungal feeding was also reported [36]. This points to alternate food choice and the ability to switch diet if root resources become limiting. Among bacterial feeders the opportunistic genera *Cephalobus* and *Acrobeloides* were fostered by litter amendment, thereby displaying an opposing trend (Fig 3). While *Cephalobus* achieved its maximum density in autumn 2012 and strongly decreased in 2013, the opposite was true for *Acrobeloides*. Likely, these taxa in the same trophic group negatively affect each other [58]. Generally, organic amendment such as mulching fosters nematode top predators and omnivores [59, 60], and comparable effects are also evident in other soil fauna, e.g. Collembola and spiders, in the detritivore food chain [61]. However, in the present study no positive impact of litter amendment was apparent at nematode higher trophic levels, rather the micro-food web structure remained disturbed and at a basal stage.

### Bulk soil food web

Under crop, i.e. with maize or wheat, the C_org_ content at the experimental field ranged between 10.6 to 13.7 mg g^-1^ soil across two growing seasons [42]. Leaving such a cropland fallow can lead to reduced soil carbon sequestration [62] and the lack of recent carbon input by crop plants at bare soil plots was expected to result in a decline in decomposer organisms and micro-food web complexity with time [28, 63]. Conform with this hypothesis, the amount of total soil PLFAs in the topsoil was generally lower in bare compared to planted soil, whereas in the rooted zone bare soil plots held an intermediate position, and no treatment effects occurred in the deeper root-free zone. This resilience of microorganisms below the plough layer suggests sufficient resources to sustain considerable biomass. The availability of maize carbon after one vegetation period was still seven times higher as compared to older carbon sources [64], pointing to maize root residues as substrate for microbial decomposition. Correspondingly, Scheunemann et al. [65] reported that soil arthropods continued to incorporate old C_3_-derived organic matter after a switch to the C_4_ crop maize. Usage of such “old” SOM by the detritivore food chain in the bulk soil likely is as an important characteristic of arable systems, where regular removal of crop cuts the internal terrestrial C-cycle, with plant carbon only partly returned to the soil as dead organic matter.

Within nematode communities certain taxa were fostered in the bare soil plots, predominantly opportunists such as the bacterial feeder *Acrobeloides* and the fungal feeder *Aphelenchoides*. The presence of these opportunists reflects the replacement of the more diverse nematode fauna under crop plant by few taxa tolerant to a wide range of environmental factors common under fallow [27]. On the other hand, the *K*-strategist *Alaimu*s, adapted to an undisturbed environment [35], increased at bare soil plots at the end of the second vegetation period. Thus with time the bare soil became a suitable habitat for certain long-lived big nematode taxa able to cope with low resource availability.

## Conclusions

The nematode micro-food web in the investigated arable soil showed marked resilience to the lack of plants, litter amendment or bare soil, and the structure of the rhizosphere, detritusphere and bulk soil food webs was little affected in the first year. In the second vegetation period, bottom-up effects maintained by belowground plant productivity were apparent and the herbivore food chain was fostered. However, neither good resource availability nor presence of both the herbivore and detritivore food chain had positive impact on higher trophic levels of the rhizosphere food web at plant plots. Comparably, only limited flux of the amended organic resources along the food chain occurred at litter plots and the detritusphere food web remained at basal conditions. Even under bare soil, with no resource input at all, the trophic structure and diversity of the bulk soil food web was only moderately affected. Instead detritivores efficiently exploited organic resources from previous vegetation periods, with the establishment of long-lived *K*-strategists pointing to stable environmental conditions. In sum, the different decomposer food webs in the arable soil were well adapted to changes in carbon availability and major pools. Apparently, the turnover of organic resources from previous vegetation period was sufficient to support the demands of the micro-food web for at least two growing seasons.

## Supporting information

S1 Table Volumetric water content in soil cropped with maize (plant), amended with maize shoot litter (litter) or bare soil along the depth profile. Presented are the average values (yearly quarter) in different seasons in 2012 and 2013. Statistical significances are based on two-way ANOVA with the factors season (S) and treatment (T); ***—*P*<0.001.
(DOCX)

S2 Table Non-parametric Spearman correlation between nematode trophic groups and the biomass of different microbial food sources. A—Gram-positive bacteria, B—Gram-negative bacteria, C—fungi. Investigated were the topsoil (0–10 cm), rooted zone (40–50 cm) and root free zone (60–70 cm) in two successive years. Significant correlations are marked in bold.–trophic group or diet not present.
(DOCX)
