# Supplementary material for: Disentangling the root- and detritus-based food chain in the micro-food web of an arable soil by plant removal
Source: PLoS One. 2017 Jul 13;12(7):e0180264. doi: 10.1371/journal.pone.0180264 (PMC5509179; doi:10.1371/journal.pone.0180264)
Supplement: S1 Table — Presented are the average values (yearly quarter) in different seasons in 2012 and 2013. Statistical significances are based on two-way ANOVA with the factors season (S) and treatment (T); ***—P<0.001. (DOCX) [file pone.0180264.s001.docx]

**Table S1**

| Depth | **Autumn 2012** | | | **Winter 2012** | | | **Summer 2013** | | | ANOVA |
| --- | --- | --- | --- | --- | --- | --- | --- | --- | --- | --- |
|  | Plant | Litter | Bare soil | Plant | Litter | Bare soil | Plant | Litter | Bare soil |  |
|  |  |  |  |  |  |  |  |  |  |  |
| 0-10 cm | 27.5 ± 2.3 | 30.8 ± 1.9 | 29.5 ± 1.6 | 31.9 ± 2.9 | 30.2 ± 1.4 | 31.6 ± 1.4 | 29.8 ± 4.6 | 24.2 ± 4.1 | 25.7 ± 4.5 | T***, S***, TxS*** |
| 10-20 cm | 25.5 ± 1.0 | 28.5 ± 0.8 | 28.5 ± 1.3 | 28.3 ± 1.8 | 28.7 ± 1.2 | 29.3 ± 1.2 | 31.8 ± 1.4 | 30.2 ± 1.9 | 29.7 ± 1.5 | T***, S***, TxS*** |
| 20-30 cm | 27.3 ± 1.8 | 30.1 ± 0.7 | 29.0 ± 1.1 | 29.0 ± 2.1 | 29.7 ± 0.8 | 29.4 ± 0.8 | 35.4 ± 1.2 | 30.3 ± 1.3 | 29.6 ± 1.3 | T***, S***, TxS*** |
| 40-50 cm | 25.8 ± 0.5 | 31.4 ± 0.8 | 29.1 ± 1.1 | 27.7 ± 2.2 | 30.6 ± 0.9 | 29.2 ± 0.7 | 33.6 ± 0.9 | 32.8 ± 1.4 | 29.6 ± 1.3 | T***, S***, TxS*** |
