# Supplementary material for: Disentangling the root- and detritus-based food chain in the micro-food web of an arable soil by plant removal
Source: PLoS One. 2017 Jul 13;12(7):e0180264. doi: 10.1371/journal.pone.0180264 (PMC5509179; doi:10.1371/journal.pone.0180264)
Supplement: S2 Table — A—Gram-positive bacteria, B—Gram-negative bacteria, C—fungi. Investigated were the topsoil (0–10 cm), rooted zone (40–50 cm) and root free zone (60–70 cm) in two successive years. Significant correlations are marked in bold.–trophic group or diet not present. (DOCX) [file pone.0180264.s002.docx]

**Table S 2A**

| **Depth** | **Trophic group** | **Gram-positive bacteria** | | | | | | | | | | | |
| --- | --- | --- | --- | --- | --- | --- | --- | --- | --- | --- | --- | --- | --- |
|  |  | **Summer 2012** | | **Autumn 2012** | | **Winter 2012** | | **Summer 2013** | | **Autumn 2013** | | **Winter 2013** | |
|  |  | ***R*** | ***P*** | ***R*** | ***P*** | ***R*** | ***P*** | ***R*** | ***P*** | ***R*** | ***P*** | ***R*** | ***P*** |
| 0-10 cm | Bacterial feeders | 0.46 | 0.13 | -0.10 | 0.75 | 0.10 | 0.76 | -0.06 | 0.85 | 0.24 | 0.44 | -0.48 | 0.12 |
|  | Plant feeders | 0.51 | 0.09 | -0.02 | 0.95 | -0.01 | 0.97 | 0.10 | 0.75 | 0.53 | 0.08 | 0.09 | 0.78 |
|  | Fungal feeders | -0.43 | 0.16 | -0.15 | 0.63 | 0.17 | 0.60 | 0.20 | 0.54 | 0.08 | 0.81 | -0.32 | 0.31 |
|  | Predators | 0.09 | 0.79 | -0.71 | 0.01 | 0.15 | 0.64 | 0.44 | 0.15 | 0.31 | 0.32 | -0.25 | 0.43 |
|  | Omnivores | 0.42 | 0.17 | -0.03 | 0.92 | 0.01 | 0.98 | 0.18 | 0.57 | -0.27 | 0.40 | **0.59** | **0.04** |
|  |  |  |  |  |  |  |  |  |  |  |  |  |  |
| 40-50 cm | Bacterial feeders | **0.93** | **0.00** | **0.59** | **0.04** | 0.13 | 0.70 | -0.24 | 0.44 | **0.61** | **0.04** | **0.72** | **0.01** |
|  | Plant feeders | **0.83** | **0.00** | **0.76** | **0.00** | **0.60** | **0.04** | 0.20 | 0.54 | 0.43 | 0.16 | **0.73** | **0.01** |
|  | Fungal feeders | 0.55 | 0.06 | 0.40 | 0.20 | 0.30 | 0.34 | 0.04 | 0.90 | 0.48 | 0.11 | **0.80** | **0.00** |
|  | Predators | 0.41 | 0.18 | - | - | -0.13 | 0.68 | -0.22 | 0.50 | 0.48 | 0.11 | - | - |
|  | Omnivores | **0.76** | **0.00** | **0.70** | **0.01** | 0.34 | 0.28 | - | - | 0.15 | 0.64 | - | - |
|  |  |  |  |  |  |  |  |  |  |  |  |  |  |
| 60-70 cm | Bacterial feeders | 0.47 | 0.12 | -0.18 | 0.57 | 0.57 | 0.05 | -0.01 | 0.98 | 0.34 | 0.28 | 0.10 | 0.75 |
|  | Plant feeders | 0.50 | 0.10 | 0.41 | 0.18 | 0.20 | 0.53 | 0.30 | 0.35 | **0.59** | **0.04** | 0.02 | 0.94 |
|  | Fungal feeders | 0.38 | 0.23 | 0.06 | 0.85 | 0.10 | 0.76 | -0.38 | 0.23 | 0.10 | 0.75 | 0.21 | 0.51 |
|  | Predators | - | - | - | - | 0.53 | 0.07 | - | - | -0.31 | 0.33 | - | - |
|  | Omnivores | -0.04 | 0.89 | -0.22 | 0.50 | -0.18 | 0.57 | - | - | -0.28 | 0.39 | 0.04 | 0.89 |

**Table S2B**

| **Depth** | **Trophic group** | **Gram-negative bacteria** | | | | | | | | | | | |
| --- | --- | --- | --- | --- | --- | --- | --- | --- | --- | --- | --- | --- | --- |
|  |  | **Summer 2012** | | **Autumn 2012** | | **Winter 2012** | | **Summer 2013** | | **Autumn 2013** | | **Winter 2013** | |
|  |  | ***R*** | ***P*** | ***R*** | ***P*** | ***R*** | ***P*** | ***R*** | ***P*** | ***R*** | ***P*** | ***R*** | ***P*** |
| 0-10 cm | Bacterial feeders | 0.47 | 0.12 | -0.21 | 0.51 | 0.08 | 0.81 | -0.03 | 0.91 | 0.18 | 0.57 | -0.50 | 0.10 |
|  | Plant feeders | 0.47 | 0.12 | -0.03 | 0.93 | 0.01 | 0.97 | 0.15 | 0.65 | 0.57 | 0.05 | 0.06 | 0.86 |
|  | Fungal feeders | -0.40 | 0.20 | -0.19 | 0.56 | 0.32 | 0.31 | 0.15 | 0.63 | 0.06 | 0.86 | -0.17 | 0.60 |
|  | Predators | 0.12 | 0.72 | **-0.72** | **0.01** | 0.12 | 0.71 | 0.44 | 0.15 | 0.16 | 0.62 | -0.09 | 0.77 |
|  | Omnivores | 0.30 | 0.34 | 0.10 | 0.76 | 0.25 | 0.43 | 0.22 | 0.48 | -0.36 | 0.25 | **0.63** | **0.03** |
|  |  |  |  |  |  |  |  |  |  |  |  |  |  |
| 40-50 cm | Bacterial feeders | **0.94** | **0.00** | **0.64** | **0.02** | 0.08 | 0.81 | -0.16 | 0.62 | **0.61** | **0.04** | **0.77** | **0.00** |
|  | Plant feeders | **0.86** | **0.00** | **0.73** | **0.01** | **0.65** | **0.02** | 0.41 | 0.18 | 0.43 | 0.16 | **0.75** | **0.01** |
|  | Fungal feeders | 0.54 | 0.07 | 0.31 | 0.33 | 0.32 | 0.31 | -0.10 | 0.76 | 0.48 | 0.11 | **0.80** | **0.00** |
|  | Predators | 0.41 | 0.18 | - | - | -0.13 | 0.68 | -0.22 | 0.50 | 0.48 | 0.11 | - | - |
|  | Omnivores | **0.75** | **0.01** | **0.75** | **0.01** | 0.41 | 0.19 | - | - | 0.15 | 0.64 | - | - |
|  |  |  |  |  |  |  |  |  |  |  |  |  |  |
| 60-70 cm | Bacterial feeders | 0.55 | 0.07 | -0.13 | 0.68 | 0.27 | 0.40 | -0.19 | 0.56 | 0.41 | 0.19 | 0.12 | 0.71 |
|  | Plant feeders | **0.59** | **0.04** | 0.41 | 0.18 | 0.06 | 0.86 | 0.35 | 0.27 | **0.63** | **0.03** | -0.01 | 0.96 |
|  | Fungal feeders | 0.50 | 0.10 | 0.13 | 0.68 | -0.06 | 0.86 | -0.13 | 0.70 | 0.17 | 0.59 | 0.31 | 0.33 |
|  | Predators | - | - | - | - | 0.46 | 0.13 | - | - | -0.22 | 0.50 | - | - |
|  | Omnivores | -0.13 | 0.68 | -0.13 | 0.68 | -0.33 | 0.29 | - | - | -0.28 | 0.39 | 0.22 | 0.50 |

**Table S2C**

| **Depth** | **Trophic group** | **Fungi** | | | | | | | | | | | |
| --- | --- | --- | --- | --- | --- | --- | --- | --- | --- | --- | --- | --- | --- |
|  |  | **Summer 2012** | | **Autumn 2012** | | **Winter 2012** | | **Summer 2013** | | **Autumn 2013** | | **Winter 2013** | |
|  |  | ***R*** | ***P*** | ***R*** | ***P*** | ***R*** | ***P*** | ***R*** | ***P*** | ***R*** | ***P*** | ***R*** | ***P*** |
| 0-10 cm | Bacterial feeders | 0.45 | 0.14 | 0.19 | 0.56 | 0.42 | 0.17 | 0.21 | 0.51 | **0.73** | **0.01** | 0.52 | 0.08 |
|  | Plant feeders | 0.34 | 0.29 | 0.06 | 0.86 | -0.08 | 0.80 | -0.08 | 0.81 | 0.09 | 0.78 | -0.10 | 0.76 |
|  | Fungal feeders | 0.10 | 0.76 | 0.52 | 0.08 | 0.04 | 0.90 | 0.51 | 0.09 | 0.22 | 0.50 | -0.01 | 0.98 |
|  | Predators | -0.20 | 0.53 | 0.31 | 0.33 | -0.21 | 0.51 | 0.64 | 0.02 | 0.43 | 0.17 | -0.45 | 0.14 |
|  | Omnivores | 0.35 | 0.27 | 0.18 | 0.57 | 0.26 | 0.41 | -0.15 | 0.63 | 0.16 | 0.61 | **0.62** | **0.03** |
|  |  |  |  |  |  |  |  |  |  |  |  |  |  |
| 40-50 cm | Bacterial feeders | **0.75** | **0.01** | 0.38 | 0.22 | -0.10 | 0.76 | 0.15 | 0.63 | **0.59** | **0.04** | **0.69** | **0.01** |
|  | Plant feeders | **0.82** | **0.00** | 0.31 | 0.32 | **0.76** | **0.00** | 0.24 | 0.44 | 0.31 | 0.32 | **0.71** | **0.01** |
|  | Fungal feeders | 0.22 | 0.50 | 0.05 | 0.88 | 0.23 | 0.47 | 0.00 | 1.00 | 0.46 | 0.13 | **0.78** | **0.00** |
|  | Predators | 0.20 | 0.52 | - | - | -0.31 | 0.33 | -0.39 | 0.21 | 0.39 | 0.21 | - | - |
|  | Omnivores | **0.77** | **0.00** | **0.83** | **0.00** | 0.26 | 0.41 | - | - | 0.09 | 0.78 | - | - |
|  |  |  |  |  |  |  |  |  |  |  |  |  |  |
| 60-70 cm | Bacterial feeders | 0.12 | 0.71 | 0.22 | 0.50 | 0.33 | 0.30 | -0.21 | 0.51 | 0.32 | 0.31 | 0.33 | 0.30 |
|  | Plant feeders | 0.41 | 0.19 | 0.29 | 0.37 | 0.55 | 0.06 | 0.57 | 0.05 | 0.49 | 0.11 | 0.22 | 0.48 |
|  | Fungal feeders | 0.02 | 0.95 | 0.05 | 0.88 | 0.16 | 0.62 | 0.15 | 0.63 | 0.25 | 0.43 | 0.28 | 0.38 |
|  | Predators | - | - | - | - | 0.08 | 0.82 | - | - | -0.31 | 0.33 | - | - |
|  | Omnivores | -0.22 | 0.50 | -0.22 | 0.50 | 0.03 | 0.92 | - | - | 0.02 | 0.95 | -0.13 | 0.68 |
